# Identification of short open reading frames in plant genomes

**DOI:** 10.3389/fpls.2023.1094715

**Published:** 2023-02-15

**Authors:** Yong Feng, Mengyun Jiang, Weichang Yu, Jiannan Zhou

**Affiliations:** ^1^ Shenzhen Branch, Guangdong Laboratory of Lingnan Modern Agriculture, Genome Analysis Laboratory of the Ministry of Agriculture and Rural Affairs, Agricultural Genomics Institute at Shenzhen, Chinese Academy of Agricultural Sciences, Shenzhen, China; ^2^ State Key Laboratory of Crop Stress Adaptation and Improvement, School of Life Sciences, Henan University, Kaifeng, China; ^3^ Guangdong Key Laboratory of Plant Epigenetics, College of Life Sciences and Oceanography, Shenzhen University, Shenzhen, China; ^4^ Liaoning Peanut Research Institute, Liaoning Academy of Agricultural Sciences, Fuxing, China; ^5^ Key Laboratory of Tropical Fruit Biology (Ministry of Agriculture), South Subtropical Crops Research Institute, Chinese Academy of Tropical Agricultural Sciences, Zhanjiang, China

**Keywords:** small open reading frame, sORFs, ribo-seq, plant, genome

## Abstract

The roles of short/small open reading frames (sORFs) have been increasingly recognized in recent years due to the rapidly growing number of sORFs identified in various organisms due to the development and application of the Ribo-Seq technique, which sequences the ribosome-protected footprints (RPFs) of the translating mRNAs. However, special attention should be paid to RPFs used to identify sORFs in plants due to their small size (~30 nt) and the high complexity and repetitiveness of the plant genome, particularly for polyploidy species. In this work, we compare different approaches to the identification of plant sORFs, discuss the advantages and disadvantages of each method, and provide a guide for choosing different methods in plant sORF studies.

## Introduction

Short/small open reading frames (sORFs) with the capacity of encoding micropeptides shorter than 100 amino acids (aa) are widely distributed in plants, ranging from green algae (Xu et al., 2017a) to rice (Yang et al., 2021) and Arabidopsis (Hanada et al., 2007), and are engaged in various biological and molecular processes, such as plant growth, nitrogen response, symbiosis nitrogen fixation, stomatal closure, plant circadian clock, anther development, pollen tube growth, abiotic responses, plant disease resistance, morphogenesis, and growth regulation (Ong et al., 2022; Wu et al., 2022). sORFs are pervasive in plant genomes and have been detected beyond the known coding regions. According to their locations, sORFs can be classified into several groups, including uORF, dORF, lncRNA-sORFs, and intergenic-sORFs (**Table 1**). As well as in mammals and yeasts (Jeon et al., 2015; Libre et al., 2021), sORFs have also been reported in plants, and the translation of uORFs can potentially repress the translation of downstream major ORFs (mORFs) (David-Assael et al., 2005; Saul et al., 2009; Pajerowska-Mukhtar et al., 2012; Causier et al., 2022). Another function of sORFs is to mitigate the abundance of miRNAs, and therefore influence the translation of their target mRNAs. Lauressergues et al. (2015) reported two plant primary transcripts (pri-miRNAs) that contain sORFs encoding regulatory peptides, miPEP165a and miPEP171b, in *A. thaliana* and *Medicago truncatula*, respectively. Overexpressing *miPEP171b* in *M. truncatula* roots specifically improves the accumulation of endogenous mature miRNAs, resulting in a reduction in lateral root density to a similar extent as overexpression of the corresponding *pri-miR171b*. Furthermore, the peptides encoded by the sORFs, *per se*, can also be functional, involving various biological processes (reviewed in Ong et al., 2022). Given that sORFs can substantially regulate the translation of downstream ORFs and encode proteins with crucial functions, the mutation of many sORFs, particularly uORFs, would lead to dramatic phenotype changes in plants and crops. Therefore, the natural or artificial mutation of these sORFs can be used to improve vital plant processes and valuable crop traits (Xu et al., 2017b; Si et al., 2020).

**Table 1:** Categories of sORFs.

Categories	Description	Putative functions	References
uORF	sORFs located in the upstream ORF of an mRNA	Repressing main ORF translation	(Zhang et al., 2020)
CPuORF	Conserved peptide uORF	Control translation of the downstream ORF	(Jorgensen and Dorantes-Acosta, 2012)
dORF	sORFs located in the downstream ORF of an mRNA	Enhances translation of main open reading frames	(Wu et al., 2020)
lncRNA-sORFs	sORFs located in long noncoding RNAs	Plant growth	(Yang et al., 1993)
Intergenic-sORFs	sORFs located in an Intergenic region	Plant growth	(Frank and Smith, 2002)

It is noteworthy that although reports of plant sORFs are increasing dramatically, the identification of sORFs in plant genomes is still challenging. The recent advancement and application of Ribo-Seq technology have promoted the research of plant sORFs; however, the existing studies on plant sORFs are only focused on plants with simple genomes, including the model plants, Arabidopsis and rice, while investigations into sORFs in complex genomes are rare. In most, if not all, existing plant sORF studies, the methods and tools developed in mammals or yeasts were directly used to search for sORFs in plant genomes. Nevertheless, plant genomes are generally more repetitive, and many of them are polyploid or paleopolyploid. Special attention should be given to the studies of sORFs in plant genomes.

Despite the challenges and difficulties, many efforts have been made to identify sORFs from plant genomes. The first systematic study of sORFs in plants was conducted on *Arabidopsis thaliana*, where more than 7,000 sORFs were identified (Hanada et al., 2007), including 49 that induced visible phenotypic effects or that are associated with various morphological changes (Hanada et al., 2013). Subsequently, a total of 48,620 sORFs were identified in *Oryza sativa* through microarray analyses, and at least 36 were involved in Fe deficiency and excess (Bashir et al., 2014). Generally, plant sORFs can be identified using three different strategies: the conservation of coding sequences, ribosome-protected footprints, and nucleotide diversity in the natural population. In this review, we summarize the methods of sORF identification used in plant studies and discuss the challenges and caveats in plant sORF identification and possible solutions for future studies.

## sORF identification through sequence conservation

**Figure 1:**
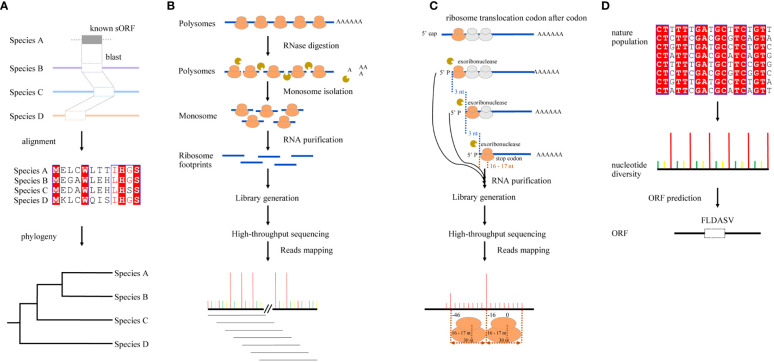
Graphic illustration for four different strategies of sORF identification. **(A)** Sequence conservation-based. It is assumed that functional sORFs are preserved by natural selection and are conserved across species; sORF identification could be based on sequence similarities using sequence alignment tools, such as BLAST. **(B)** RFP-based. The ribosome-associated mRNAs are digested by RNase and the fragments bound by ribosome are protected; they can then be isolated and sequenced using next-generation sequencing (NGS) technology. As ribosomes move along mRNA strands with a step of three nucleotides during the translation of mRNAs, the mapping loci of RPFs on mRNAs show a strong 3-nt periodicity. **(C)** Degradome sequencing-based. 5’-3’ exoribonuclease digests translating mRNAs chasing after the last translating ribosome, which translocates codon after codon on mRNAs, leaving truncated 5’ monophosphate mRNAs with a 3-nucleotide distance in length. After sequencing, this 3-nucleotide periodicity in the position of free 5’ mRNA ends can then be used for sORFs identification. **(D)** Natural nucleotide diversity-based. As only the nucleotide diversities in CDSs showed a significant 3-nt periodicity, the single-nucleotide polymorphism (SNP) datasets of natural populations can be used to predict sORFs.

As functional ORFs are conserved across genomes, early attempts at sORF identification were primarily based on sequence similarities using sequence alignment tools, such as BLAST (Altschul et al., 1997), coupled with ORF Finder (Wheeler et al., 2002), assuming that the functional sORFs would also have been preserved by natural selection (**Figure 1A**). For example, a total of 26 groups of conserved uORFs were identified in *O. sativa* and *A. thaliana* using uORF-Finder (Hayden and Jorgensen, 2007). In another study, sequence similarity was also observed at the amino acid level of uORFs across Arabidopsis and rice, 11 rice uORFs were conserved in Arabidopsis, and most of them were also conserved in other cereals (Tran et al., 2008). These conserved sORFs can be true; however, recent evidence has revealed varied degrees of sORF conservation. Although many sORFs are conserved, there are also many species- or lineage-specific sORFs (Hsu et al., 2016; Wang et al., 2021), which cannot be identified through sequence comparisons.

## sORF identification using ribosome-protected footprints

Ribo-seq is an emerging technology that enables the identification of sORFs (Ingolia, 2010). To date, most plant sORFs are identified using RPFs. Briefly, the ribosome-associated mRNAs are digested by RNase, and the fragments bound by ribosome are protected; they can then be isolated and sequenced using next-generation sequencing (NGS) technology. As ribosomes move along mRNA strands with a step of three nucleotides during the translation of mRNAs, the mapping loci of RPFs on mRNAs show a strong 3-nt periodicity, which provides information for the identification of translating frames on mRNAs (**Figure 1B**). To better analyze and mine the information in RPFs, many computation tools implementing different algorithms have been developed to predict sORFs or ORFs on noncoding RNAs (see review of Ong et al., 2022), including FLOSS (Ingolia et al., 2014), RiboTaper (Calviello et al., 2016), RiboCode (Xiao et al., 2018), ORFquant (Calviello et al., 2020), RiboNT (Song et al., 2021), and slORFfinder (Song et al., 2023). Among them, ORFScore and RiboTaper are the most frequently used tools in plant sORF studies. For example, Hsu et al. (2016) detected 187 uORFs, 10 dORFs, and 27 translated sORFs from annotated non-coding RNAs in Arabidopsis using RiboTaper (Hsu et al., 2016), and Wu et al. (2020) detected 1,406 and 1,153 dORFs in human cells and zebrafish embryos using ORFScore (Wu et al., 2020). As a caveat, it should be noted that most of these tools were originally developed in the study of mammals or yeasts, the genomes of which are much simpler than those of plants; the challenges in plant studies were not fully considered. sORF identification using RPFs is highly dependent on the accurate mapping of RPFs, and the Ribo-Seq strategy has some inherent shortcomings when it is applied in plant studies because of the short lengths of RPFs (~ 30 nt) and the complexity and repetitiveness of plant genomes. It is difficult to accurately map the short RPFs to the loci they are derived from in a complex and repetitive genome. In most of the existing studies, the solutions to this problem are either to remove the RPFs with multiple hits or to randomly retain only one of them (Hsu et al., 2016; Bazin et al., 2017; Erhard et al., 2018). However, these processes would certainly introduce errors in ORF identification, resulting in missing ORFs in the genome. Recently, a protocol profiling the footprints of two closely packed ribosomes (disomes) that can double the size of footprints was reported (Arpat et al., 2020). The RPFs of disomes (~60 nt) can somehow compromise the mapping problem caused by their short lengths; nevertheless, they are still too short to completely solve this problem, particularly in the study of polyploidy genomes. Furthermore, only ~10% of the ribosomes can be captured in disomes, with a significant bias towards rRNA and sequences encoding signal peptides; whether they can be used to identify sORFs genome-wide has not been tested. It is possible to increase the size of RPFs to ~ 90 nt by profiling the footprints of trisomes, but their representativeness should be evaluated before they can be used in sORF identification.

## sORF identification using degradome sequencing

Degradome sequencing is a high-throughput method that was originally used for the identification of endogenous siRNA and miRNA targets by combining the modified rapid amplification of 5’ cDNA ends (5’-RACE) and NGS technology (Addo-Quaye et al., 2008). Briefly, 5’-3’ exoribonuclease cleaves the translating mRNAs at the last ribosomes that can be translocated codon by codon, leaving a set of truncated transcripts with both free 5’ monophosphates and 3-nt distance in length. After being sequenced with the NGS platform, this 3-nucleotide periodicity in the position of free 5’ mRNA ends could be revealed by mapping reads to mRNAs (Bertoni, 2016), which provides an alternative approach for sORF identification (**Figure 1C**) (Hou et al., 2016). Using genome-wide mapping of truncated mRNAs, Yu et al. (2016) discovered a 3-nt periodicity pattern throughout ORFs in Arabidopsis leaf samples, and the accumulation of cleavage events at 16 to 17 nucleotides upstream of the stop codons of both ORFs and uORFs was also observed because of ribosomal pausing during translation termination. These results, therefore, make it possible to search for potential sORFs (Yu et al., 2016). In another research, 3-nt periodicity was also observed, not only in Arabidopsis but also in *Glycine max* and *Oryza sativa*, and both novel and known uORFs were identified by searching the accumulation of 5’ RNA ends peaking upstream of the stop codons (Hou et al., 2016). While degradome-based ORF prediction relies on the 3-nt periodicity of the mapping positions of NGS reads on mRNAs, truncated RNA fragments are much longer than RPFs, making them beneficial for resolving the mapping problems in complex genomes caused by short lengths of RPFs (Carpentier et al., 2021). However, as degradome sequencing was originally developed to identify the target of sRNAs, the fact that the binding of sRNAs can result in the accumulation of degradome reads out of the translating frames might, therefore, introduce unexpected errors in the prediction of ORFs. Although ORF prediction from degradome reads is similar to that from RPFs, whether those RPF-based tools can also be used to predict ORFs from degradome reads has rarely been tested. To better utilize the degradome datasets, more tools need to be developed or tested for sORF prediction in the future.

## sORF identification using nucleotide diversity

As the third nucleotides in codons are wobble nucleotides and are therefore subject to a more relaxed purification selection in nature (Hurst, 2002), resulting in higher nucleotide diversities every three nucleotides in the coding sequences (Jiang et al., 2022). This pattern resembles the 3-nt periodicity of RPFs on mRNAs and can therefore also be used to predict ORFs (**Figure 1D**). Jiang et al. (2022) recently developed a pipeline, OrfPP, to predict ORFs using the single-nucleotide polymorphisms (SNPs) datasets of natural populations and applied it to two polyploidy species: tetraploidy cotton (*Gossypium hirsutum*) and hexaploidy wheat (*Triticum aestivum*). As SNPs in most studies are usually called using 100 or 150 bp pair-ended reads, this strategy can overcome the troubles caused by the short lengths of RPFs in plant studies. Although SNP calling may also introduce several errors caused by the mismapping or multiple mapping of short reads, this problem can finally be solved by the future application of long reads in plant population studies. Indeed, long-read techniques have been used increasingly to detect genomic variants in natural populations of plants (Dorfner et al., 2022; Tang et al., 2022; Zhou et al., 2022). Another advantage of this method is the direct use of existing datasets (SNPs) requiring no extra experiments, such as the construction of Ribo-Seq libraries, which could be costly and technically challenging in some organisms or tissues, therefore allowing the large-scale identification of sORFs in plants with genome-wide SNP datasets. The SNP-based strategy can be used in complex genomes as long as their SNP datasets are available. Nevertheless, genome sequencing and assembly are also challenging and costly for these species, many of which do not have genome-wide SNP datasets either. The lack of SNPs prevents the application of an SNP-based approach in these species. Fortunately, for most polyploidy crops, such as oilseed, cotton, and wheat, the reference genome assembly and population resequencing have been completed, which can facilitate the identification of sORFs using the SNP-based approach.

## Discussion

Although several approaches have been developed to identify sORFs, each has its own limitations, and caution should be taken in the application of different methods. Sequence conservation-based methods can only identify old and conserved sORFs but are powerless in identifying young sORFs. RPFs can only make use of short reads, thus resulting in incorrect mapping to genomes, and this problem would be even worse in a complex and repetitive genome. The degradome and SNP-based approaches take advantage of longer reads to produce high-quality unique mapping and should lead to better sORF prediction. Both the RPF and degradome-based identification are affected by the reads captured in the experiments, so the silent or lowly translated sORFs might have been missed in these data and the results can be substantially varied across different tissues or growth conditions. In contrast, the SNP-based strategy relies on the preparation of a high-quality library and can predict both active and inactive sORFs. Cross-identification using different approaches was also reported. Of the 89 conserved Arabidopsis sORFs, 39 were successfully identified by RPFs (Hsu et al., 2016). More than a quarter of the sORFs predicted from SNPs, which were actively translated, overlapped with those predicted using RPFs (Jiang et al., 2022). Thus, the SNP-based strategy is an effective approach to extending the study of sORFs, especially in complex genomes, but it requires the accumulation of nucleotide diversity in natural populations, and accuracy is also affected by the quality of the reference genome and SNPs datasets. In practice, these approaches are mutually complementary and can be chosen for different purposes. Overall, it has become increasingly clear that sORFs play important roles in various plant processes and are potential candidates for crop improvement. Although the application of Ribo-Seq in plant studies has substantially enhanced the understanding of plant sORFs, most of these studies were conducted in model species, such as Arabidopsis (Hsu et al., 2016; Mahboubi et al., 2021) and rice (Su et al., 2018; Xu et al., 2021), or crops with small and simple genomes, such as tomato (Wu et al., 2019); the sORFs in other plants, particularly polyploidy species, are poorly investigated. Given that approximately 47–70% of angiosperm species are polyploid (Masterson, 1994), more advanced techniques and algorithms are required in the future to enhance the understanding of plant sORFs.

## Author contributions

JZ and WY conceived the idea. YF and MJ performed the literature search and data collection. YF and JZ wrote the manuscript. JZ and WY revised the manuscript. All authors contributed to the article and approved the submitted version.

## References

[B1] Addo-Quaye C. Eshoo T. W. Bartel D. P. Axtell M. J. (2008). Endogenous siRNA and miRNA targets identified by sequencing of the arabidopsis degradome. Curr. Biol. 18, 758–762. doi: 10.1016/j.cub.2008.04.042 18472421 PMC2583427

[B2] Altschul S. F. Madden T. L. Schäffer A. A. Zhang J. Zhang Z. Miller W.. (1997). Gapped BLAST and PSI-BLAST: a new generation of protein database search programs. Nucleic Acids Res. 25, 3389–3402. doi: 10.1093/nar/25.17.3389 9254694 PMC146917

[B3] Arpat A. B. Liechti A. De Matos M. Dreos R. Janich P. Gatfield D. (2020). Transcriptome-wide sites of collided ribosomes reveal principles of translational pausing. Genome Res. 30, 985–999. doi: 10.1101/gr.257741.119 32703885 PMC7397865

[B4] Bashir K. Hanada K. Shimizu M. Seki M. Nakanishi H. Nishizawa N. K. (2014). Transcriptomic analysis of rice in response to iron deficiency and excess. Rice 7, 18. doi: 10.1186/s12284-014-0018-1 26224551 PMC4884027

[B5] Bazin J. Baerenfaller K. Gosai S. J. Gregory B. D. Crespi M. Bailey-Serres J. (2017). Global analysis of ribosome-associated noncoding RNAs unveils new modes of translational regulation. Proc. Natl. Acad. Sci. 114, E10018–E10027. doi: 10.1073/pnas.1708433114 29087317 PMC5699049

[B6] Bertoni G. (2016). RNA Degradome studies give insights into ribosome dynamics. Plant Cell 28, 2348–2349. doi: 10.1105/tpc.16.00785 27754877 PMC5134993

[B7] Calviello L. Hirsekorn A. Ohler U. (2020). Quantification of translation uncovers the functions of the alternative transcriptome. Nat. Struct. Mol. Biol. 27, 717–725. doi: 10.1038/s41594-020-0450-4 32601440

[B8] Calviello L. Mukherjee N. Wyler E. Zauber H. Hirsekorn A. Selbach M.. (2016). Detecting actively translated open reading frames in ribosome profiling data. Nat. Methods 13, 165–170. doi: 10.1038/nmeth.3688 26657557

[B9] Carpentier M.-C. Bousquet-Antonelli C. Merret R. (2021). Fast and efficient 5′P degradome library preparation for analysis of Co-translational decay in arabidopsis. Plants 10, 466. 33804539 10.3390/plants10030466 PMC7998949

[B10] Causier B. Hopes T. McKay M. Paling Z. Davies B. (2022). Plants utilise ancient conserved peptide upstream open reading frames in stress-responsive translational regulation. Plant Cell Environ. 45, 1229–1241. doi: 10.1111/pce.14277 35128674 PMC9305500

[B11] David-Assael O. Saul H. Saul V. Mizrachy-Dagri T. Berezin I. Brook E.. (2005). Expression of AtMHX, an arabidopsis vacuolar metal transporter, is repressed by the 5' untranslated region of its gene. J. Exp. Bot. 56, 1039–1047. doi: 10.1093/jxb/eri097 15710632

[B12] Dorfner M. Ott T. Ott P. Oberprieler C. (2022). Long-read genotyping with SLANG (Simple long-read loci assembly of nanopore data for genotyping). Appl. Plant Sci. 10, e11484. doi: 10.1002/aps3.11484 35774992 PMC9215276

[B13] Erhard F. Halenius A. Zimmermann C. L'Hernault A. Kowalewski D. J. Weekes M. P.. (2018). Improved ribo-seq enables identification of cryptic translation events. Nat. Methods 15, 363–366. doi: 10.1038/nmeth.4631 29529017 PMC6152898

[B14] Frank M. J. Smith L. G. (2002). A small, novel protein highly conserved in plants and animals promotes the polarized growth and division of maize leaf epidermal cells. Curr. Biol. 12, 849–853. 12015123 10.1016/s0960-9822(02)00819-9

[B15] Hanada K. Higuchi-Takeuchi M. Okamoto M. Yoshizumi T. Shimizu M. Nakaminami K.. (2013). Small open reading frames associated with morphogenesis are hidden in plant genomes. Proc. Natl. Acad. Sci. 110, 2395–2400. doi: 10.1073/pnas.1213958110 23341627 PMC3568369

[B16] Hanada K. Zhang X. Borevitz J. O. Li W.-H. Shiu S.-H. (2007). A large number of novel coding small open reading frames in the intergenic regions of the *Arabidopsis thaliana* genome are transcribed and/or under purifying selection. Mol. Plant-Microbe Interact. 17, 632–640. doi: 10.1101/gr.5836207 PMC1855179 17395691

[B17] Hayden C. A. Jorgensen R. A. (2007). Identification of novel conserved peptide uORF homology groups in *Arabidopsis* and rice reveals ancient eukaryotic origin of select groups and preferential association with transcription factor-encoding genes. BMC Biol. 5, 32. doi: 10.1186/1741-7007-5-32 17663791 PMC2075485

[B18] Hou C. Y. Lee W. C. Chou H. C. Chen A. P. Chou S. J. Chen H. M. (2016). Global analysis of truncated RNA ends reveals new insights into ribosome stalling in plants. Plant Cell 28, 2398–2416. doi: 10.1105/tpc.16.00295 27742800 PMC5134977

[B19] Hsu P. Y. Calviello L. Wu H.-Y. L. Li F.-W. Rothfels C. J. Ohler U.. (2016). Super-resolution ribosome profiling reveals unannotated translation events in arabidopsis. Proc. Natl. Acad. Sci. 113, E7126–E7135. doi: 10.1073/pnas.1614788113 27791167 PMC5111709

[B20] Hurst L. D. (2002). The Ka/Ks ratio: diagnosing the form of sequence evolution. Trends Genet. 18, 486–487. doi: 10.1016/S0168-9525(02)02722-1 12175810

[B21] Ingolia N. T. (2010). Chapter 6 - genome-wide translational profiling by ribosome footprinting. Methods Enzymol. 470, 119–142. doi: 10.1016/S0076-6879(10)70006-9 20946809

[B22] Ingolia N. T. Brar Gloria A. Stern-Ginossar N. Harris Michael S. Talhouarne Gaëlle J. S. Jackson Sarah E.. (2014). Ribosome profiling reveals pervasive translation outside of annotated protein-coding genes. Cell Rep. 8, 1365–1379. doi: 10.1016/j.celrep.2014.07.045 25159147 PMC4216110

[B23] Jeon S. Lim S. Ha J. Kim J. (2015). Identification of Psk2, Skp1, and Tub4 as trans-acting factors for uORF-containing ROK1 mRNA in saccharomyces cerevisiae. J. Microbiol. 53, 616–622. doi: 10.1007/s12275-015-5389-5 26310304

[B24] Jiang M. Ning W. Wu S. Wang X. Zhu K. Li A.. (2022). Three-nucleotide periodicity of nucleotide diversity in a population enables the identification of open reading frames. Briefings Bioinf. 23 (4), bbac210. doi: 10.1093/bib/bbac210 PMC9294425 35698834

[B25] Jorgensen R. Dorantes-Acosta A. (2012). Conserved peptide upstream open reading frames are associated with regulatory genes in angiosperms, Vol. 2012. 3. 10.3389/fpls.2012.00191 PMC3426882 22936940

[B26] Lauressergues D. Couzigou J.-M. Clemente H. S. Martinez Y. Dunand C. Bécard G.. (2015). Primary transcripts of microRNAs encode regulatory peptides. Nature 520, 90–93. 25807486 10.1038/nature14346

[B27] Libre C. Seissler T. Guerrero S. Batisse J. Verriez C. Stupfler B.. (2021). Paillart JC. a conserved uORF regulates APOBEC3G translation and is targeted by HIV-1 vif protein to repress the antiviral factor. Biomedicines 10 (1). doi: 10.3390/biomedicines10010013 PMC8773096 35052693

[B28] Mahboubi A. Delhomme N. Häggström S. Hanson J. (2021). Small-scale sequencing enables quality assessment of ribo-seq data: an example from arabidopsis cell culture. Plant Methods 17, 92. doi: 10.1186/s13007-021-00791-w 34429136 PMC8386038

[B29] Masterson J. (1994). Stomatal size in fossil plants: Evidence for polyploidy in majority of angiosperms. Science 264, 421–424. doi: 10.1126/science.264.5157.421 17836906

[B30] Ong S. N. Tan B. C. Al-Idrus A. Teo C. H. (2022). Small open reading frames in plant research: from prediction to functional characterization. 3 Biotech. 12, 76. doi: 10.1007/s13205-022-03147-w PMC8873315 35251879

[B31] Pajerowska-Mukhtar K. M. Wang W. Tada Y. Oka N. Tucker Chandra L. Fonseca Jose P.. (2012). The HSF-like transcription factor TBF1 is a major molecular switch for plant growth-to-Defense transition. Curr. Biol. 22, 103–112. doi: 10.1016/j.cub.2011.12.015 22244999 PMC3298764

[B32] Saul H. Elharrar E. Gaash R. Eliaz D. Valenci M. Akua T.. (2009). The upstream open reading frame of the *Arabidopsis* AtMHX gene has a strong impact on transcript accumulation through the nonsense-mediated mRNA decay pathway. Plant J. 60, 1031–1042. doi: 10.1111/j.1365-313X.2009.04021.x 19754518

[B33] Si X. Zhang H. Wang Y. Chen K. Gao C. (2020). Manipulating gene translation in plants by CRISPR–Cas9-mediated genome editing of upstream open reading frames. Nat. Protoc. 15, 338–363. doi: 10.1038/s41596-019-0238-3 31915386

[B34] Song B. Jiang M. Gao L. (2021). RiboNT: A noise-tolerant predictor of open reading frames from ribosome-protected footprints. Life 11, 701. doi: 10.3390/life11070701 34357073 PMC8307163

[B35] Song B. Li H. Jiang M. Gao Z. Wang S. Gao L.. (2023). slORFfinder: a tool to detect open reading frames resulting from trans-splicing of spliced leader sequences. Briefings Bioinf. 24 (1), bbac610. doi: 10.1093/bib/bbac610 PMC9851317 36611257

[B36] Su Z. Tang Y. Ritchey L. E. Tack D. C. Zhu M. Bevilacqua P. C.. (2018). Genome-wide RNA structurome reprogramming by acute heat shock globally regulates mRNA abundance. Proc. Natl. Acad. Sci. 115, 12170–12175. doi: 10.1073/pnas.1807988115 30413617 PMC6275526

[B37] Tang D. Jia Y. Zhang J. Li H. Cheng L. Wang P.. (2022). Genome evolution and diversity of wild and cultivated potatoes. Nature 606, 535–541. doi: 10.1038/s41586-022-04822-x 35676481 PMC9200641

[B38] Tran M. K. Schultz C. J. Baumann U. (2008). Conserved upstream open reading frames in higher plants. BMC Genomics 9, 361. doi: 10.1186/1471-2164-9-361 18667093 PMC2527020

[B39] Wang B. Hao J. Pan N. Wang Z. Chen Y. Wan C. (2021). Identification and analysis of small proteins and short open reading frame encoded peptides in Hep3B cell. J. Proteomics 230, 103965. doi: 10.1016/j.jprot.2020.103965 32891891

[B40] Wheeler D. L. Church D. M. Lash A. E. Leipe D. D. Madden T. L. Pontius J. U.. (2002). Database resources of the national center for biotechnology information: 2002 update. Nucleic Acids Res. 30, 13–16. doi: 10.1093/nar/30.1.13 11752242 PMC99094

[B41] Wu H.-W. Fajiculay E. Wu J.-F. Yan C.-C. S. Hsu C.-P. Wu S.-H. (2022). Noise reduction by upstream open reading frames. Nat. Plants 8 (5), 474–480. doi: 10.1038/s41477-022-01136-8 PMC9122824 35501454

[B42] Wu H.-Y. L. Song G. Walley J. W. Hsu P. Y. (2019). The tomato translational landscape revealed by transcriptome assembly and ribosome Profiling1 [OPEN]. Plant Physiol. 181, 367–380. doi: 10.1104/pp.19.00541 31248964 PMC6716236

[B43] Wu Q. Wright M. Gogol M. M. Bradford W. D. Zhang N. Bazzini A. A. (2020). Translation of small downstream ORFs enhances translation of canonical main open reading frames. EMBO J. 39, e104763. doi: 10.15252/embj.2020104763 32744758 PMC7459409

[B44] Xiao Z. Huang R. Xing X. Chen Y. Deng H. Yang X. (2018). *De novo* annotation and characterization of the translatome with ribosome profiling data. Nucleic Acids Res. 46, e61–e61. doi: 10.1093/nar/gky179 29538776 PMC6007384

[B45] Xu G. Greene G. H. Yoo H. Liu L. Marqués J. Motley J.. (2017a). Global translational reprogramming is a fundamental layer of immune regulation in plants. Nature 545, 487–490. doi: 10.1038/nature22371 28514447 PMC5485861

[B46] Xu Q. Liu Q. Chen Z. Yue Y. Liu Y. Zhao Y.. (2021). Histone deacetylases control lysine acetylation of ribosomal proteins in rice. Nucleic Acids Res. 49, 4613–4628. doi: 10.1093/nar/gkab244 33836077 PMC8096213

[B47] Xu G. Yuan M. Ai C. Liu L. Zhuang E. Karapetyan S.. (2017b). uORF-mediated translation allows engineered plant disease resistance without fitness costs. Nature 545, 491–494. doi: 10.1038/nature22372 28514448 PMC5532539

[B48] Yang W. C. Katinakis P. Hendriks P. Smolders A. de Vries F. W. Spee J. H.. (1993). HJJTPjfc, biology m. characterization of GmENOD40, a gene showing novel patterns of cell-specific expression during soybean nodule development. Plant J. 3 (4), 573–585. 8220464 10.1046/j.1365-313x.1993.03040573.x

[B49] Yang X. Song B. Cui J. Wang L. Wang S. Luo L.. (2021). Comparative ribosome profiling reveals distinct translational landscapes of salt-sensitive and -tolerant rice. BMC Genomics 22, 612. doi: 10.1186/s12864-021-07922-6 34384368 PMC8359061

[B50] Yu X. Willmann M. R. Anderson S. J. Gregory B. D. (2016). Genome-wide mapping of uncapped and cleaved transcripts reveals a role for the nuclear mRNA cap-binding complex in cotranslational RNA decay in arabidopsis. Plant Cell 28, 2385–2397. doi: 10.1186/s12864-021-07922-6 27758893 PMC5134982

[B51] Zhang T. Wu A. Yue Y. Zhao Y. (2021). uORFs: Important cis-regulatory elements in plants, Vol. 21. 6238. 10.3390/ijms21176238 PMC7503886 32872304

[B52] Zhou Y. Zhang Z. Bao Z. Li H. Lyu Y. Zan Y.. (2022). Graph pangenome captures missing heritability and empowers tomato breeding. Nature 606, 527–534. doi: 10.1038/s41586-022-04808-9 35676474 PMC9200638

